# Hemostatic effect and psychological impact of an oxidized regenerated cellulose patch after transrectal ultrasound-guided prostate biopsy: A prospective and retrospective study

**DOI:** 10.1097/MD.0000000000015623

**Published:** 2019-05-17

**Authors:** Ji Woon Park, Jung Im Kim, Sang Rak Bae, Yong Seok Lee, Chang Hee Han, Sung Hak Kang, Bong Hee Park

**Affiliations:** a Department of Urology, Uijeongbu St. Mary's Hospital, College of Medicine, The Catholic University of Korea; b Department of Radiology, Kyung Hee University Hospital at Gangdong, Kyung Hee University College of Medicine, Seoul, Republic of Korea.

**Keywords:** anxiety, biopsy, bleeding, prostate, surgicel

## Abstract

To investigate the usefulness of the oxidized regenerated cellulose patch (ORCP) for postbiopsy hemostasis, anxiety, and depression in patients undergoing transrectal ultrasound-guided prostate biopsy.

This was a prospective-retrospective study of 300 patients who underwent systematic 12-core prostate biopsy from August 2016 through March 2018. The ORCP was inserted into the rectum immediately after prostate biopsy in the prospective group (n = 150), while the retrospective group (n = 150) underwent prostate biopsy alone. The frequency rate and duration of hematuria, rectal bleeding, and hematospermia were compared between the 2 groups. Anxiety and depression were assessed with the hospital anxiety and depression scale before and after prostate biopsy in the prospective group.

The frequency rates of hematuria and hematospermia showed no significant differences between the prospective versus retrospective groups (64.7% vs 66.7%, *P* = .881; 18 vs 20%, *P* = .718; respectively). Frequency of rectal bleeding was significantly lower in the prospective group than in the retrospective group (26.7% vs 42.7%, *P* = .018). However, there were no significant differences in median duration of rectal bleeding, hematuria, or hematospermia between the 2 groups (2, 5, and 2 days vs 2, 7, and 1 day, *P* > .05, respectively, for the prospective vs retrospective group). Multivariate analysis found that ORCP insertion was a significant protective factor against postbiopsy rectal bleeding (*P* = .038, odds ratio 0.52). Only anxiety level in the prospective group before versus after prostate biopsy was significantly reduced (5 vs 4, *P* = .011).

ORCP insertion after prostate biopsy is an effective and simple method for decreasing rectal bleeding. ORCP insertion may also alleviate anxiety in patients undergoing prostate biopsy.

## 1 Introduction

Transrectal ultrasound (TRUS)-guided prostate biopsy is the standard method for histopathological diagnosis of prostate cancer (PCa). With the common practice of prostate-specific antigen (PSA) testing, TRUS-guided prostate biopsy has become one of the most frequently performed urological procedures in the outpatient setting.^[1]^ Although the procedure is generally considered safe and well-tolerated, complications can occur. Postbiopsy bleeding including hematospermia, hematuria, and rectal bleeding is the most frequent and troublesome complication associated with TRUS-guided prostate biopsy, but it is commonly self-limiting and minor.^[2]^ Although excessive rectal bleeding is infrequent, it can be a potentially life-threatening complication. However, there is no consensus about routine procedures for preventing or decreasing bleeding complications following prostate biopsy.

Only a few reports have suggested methods for reducing hemorrhagic complications following prostate biopsy, such as rectal Foley catheterization, insertion of a gelatin sponge into the rectum, and ultrasound-guided compression on a bleeding biopsy tract by ultrasound transducer.^[3–5]^ Topical hemostatic agents have commonly been used to control bleeding in various urologic procedures.^[6,7]^ Of these, oxidized regenerated cellulose patch (ORCP) is well known and accepted due to its favorable biocompatibility, ease of use, and bactericidal property.^[8]^ There is no study assessing the efficacy of ORCP on hemostasis in prostate biopsy.

Anxiety and/or depression levels are important factors to consider in invasive procedures such as prostate biopsy. Macefield et al^[9]^ reported that about 20% of patients experienced anxious moods and high stress when undergoing TRUS-guided prostate biopsy. Psychological stress such as anxiety and depression may interfere with invasive examination. There are few reports on strategies to relieve psychological distress in patients undergoing prostate biopsy. Owing to the significance of early detection of PCa and increasing acceptance of active surveillance, the number of prostate biopsies likely will increase.^[10]^ Therefore, it is critical to reduce postbiopsy complications and the associated anxiety and depression levels.

The objective of this study was to investigate the efficacy of immediate ORCP insertion on postbiopsy bleeding after prostate biopsy. Additionally, we evaluated the impact of immediate ORCP insertion after prostate biopsy on anxiety and depression levels of patients undergoing the procedure.

## 2 Materials and methods

### 2.1 Study population

This was a prospective-retrospective cohort study performed from August 2016 through March 2018, with approval of the Institutional Review Board of the Catholic University of Korea. A total of 189 patients who underwent prostate biopsy from August 2016 through May 2017 were recruited into the retrospective cohort (Group I). The prospective cohort (Group II) comprised 196 patients who received prostate biopsy between June 2017 and March 2018. The indications for prostate biopsy were PSA value greater than 4.0 ng/mL and/or abnormal digital rectal examination. The exclusion criteria included a history of previous prostatic biopsy, < or > 12 biopsy cores, concurrent use of anticoagulation or antiplatelet drugs, coagulation disorder and conditions, such as bladder or renal tumors, hemorrhoids, urinary tract calculi, rectal inflammatory diseases or anal fissure, which could potentially cause hematuria or rectal bleeding and thus interfere with bleeding evaluation. All participants were advised to discontinue any antiplatelet or anticoagulant agents for at least 1 week before the prostate biopsy. Written informed consents was obtained from all participants.

### 2.2 Biopsy protocol

Prophylactic oral quinolone was given before and continually after prostate biopsy (for a total duration of 1 week), and aminoglycoside was intramuscularly injected just before the procedure. No local anesthesia was administered to prevent interference with bleeding. All prostate biopsies were conducted in the outpatient setting by 1 urologist. TRUS examination was performed with the patient in the left decubitus position using a 7.5 MHz biplane probe (Prosound SSD-3500; Aloka, Tokyo, Japan). Systematic 12-core prostate biopsy was performed using an automatic biopsy gun with an 18-gauge needle (Bard Magnum; Bard Medical, Covington). In only group II, ORCP (Surgicel Original; Ethicon, New Brunswick) was inserted into the rectum with finger guidance immediately after TRUS-guided prostate biopsy.

### 2.3 Morbidity assessment

All patients were evaluated 7 days following prostate biopsy to discuss the pathological results of biopsy and any complications. Two nonvalidated questionnaires were used to evaluate patient characteristics and morbidities associated with the procedure. The first questionnaire comprised variables of age, body mass index (BMI), hypertension, diabetes mellitus (DM), American Society of Anesthesiologists (ASA) score, prostate volume, PSA value, and any immediate complications. This questionnaire was administered by the urologist who conducted the prostate biopsy. The second questionnaire included questions about the presence and duration of postbiopsy bleeding, occurrence of other complications, and the use of any medical service. Regarding complications asked about in the second questionnaire, another urologist evaluated by telephone interview at 14 days and 4 weeks after prostate biopsy. As of March 2015, the institution decided to administer this morbidity assessment protocol to all patients undergoing prostate biopsy. Hematuria was defined as grossly visible bleeding in the urine, hematospermia was defined as evident bleeding in the semen and rectal bleeding was defined as spontaneous or defecation-related bleeding from the rectum. Pain during biopsy was determined with the visual analog scale (VAS) from 0 (no pain) to 10 (the worst pain imaginable). Bleeding complications were categorized as mild (eg, self-limited hematuria, hematospermia, rectal bleeding) or severe (eg, hematuria, hematospermia, or rectal bleeding requiring transfusion, hospitalization, or any type of medical intervention).^[11]^

### 2.4 Psychological assessment

Anxiety and depression were prospectively assessed with the hospital anxiety and depression scale (HADS) in group II.^[12]^ The HADS consists of 7 items to evaluate anxiety symptoms and 7 items to evaluate depressive symptoms. Each item is rated on a 4-point scale scored 0 to 3. The scores for anxiety and depression range between 0 and 21 (0–7 normal, 8–10 borderline abnormal, 11–21 abnormal). The participants were informed about the procedure and potential complications, and then asked to fill out the HADS questionnaires before prostate biopsy. The participants were informed about ORCP insertion for prevention of bleeding complications and the same questionnaires were repeated after prostate biopsy.

### 2.5 Statistical analyses

Continuous data (median/interquartile range [IQR]) were compared with the independent *t* test or Mann–Whitney *U* test and categorical data (absolute value/percentage) were compared with the chi-square or Fisher exact test. Univariable and multivariable logistic regression analyses were carried out to determine predictors associated with rectal bleeding. Paired *t* test was used to compare changes in HADS score. Statistical analyses were performed with SPSS, version 13.0 (IBM, Armonk) and *P* < .05 indicated a significant difference.

## 3 Results

**Figure 1:**
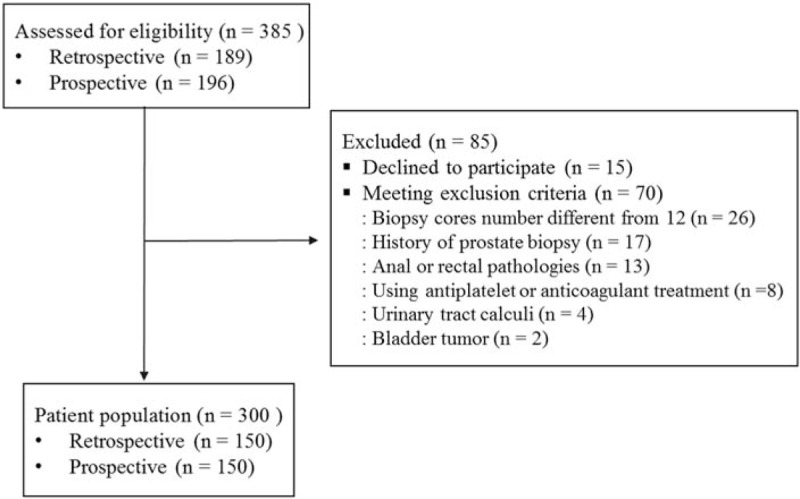
Flow chart of patients who met inclusion and exclusion criteria.

In total, 300 patients were enrolled (Fig. 1). Table 1 shows the clinicopathologic characteristics of the 2 groups. The median age was 70 years (IQR 62–74). The median prostate volume was 41.6 mL (IQR 32.5–56.8), and the median PSA value was 6.8 ng/mL (IQR 4.8–11.3). The overall detection rate of PCa was 33.0% (99/300). Age, ASA score, BMI, presence of hypertension or DM, prostate volume, PSA level, and detection rate of PCa were comparable between the groups.

**Table 1:**
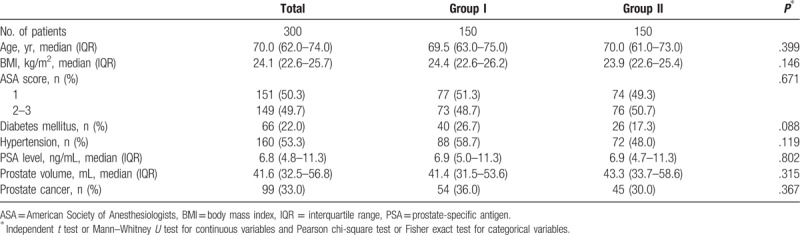
Characteristics of patients who underwent transrectal ultrasound-guided prostate biopsy.

Hematuria was the most common complication after prostate biopsy in the 2 groups. There were no statistical differences between the 2 groups in frequency and median duration of hematuria (I vs II, 66.7% vs 64.7%, *P* = .881; 2.0 vs 2.0 days, *P* = .327). The median duration for rectal bleeding in Groups I and II were similar (I vs II, 1.0 vs 2.0 days, *P* = .333). However, rectal bleeding rates were significantly lower in Group II than in Group I (42.7% vs 26.7%, *P* = .018). There were no significant differences between the 2 groups in the frequency and median duration of hematospermia (I vs II, 20% vs 18%, *P* = .718; 7.0 vs 5.0 days, *P* = .388). The median VAS score was 4.0 in the 2 groups, with no significant difference (*P* = .885). The rates of other complication after prostate biopsy (lower urinary tract symptom [LUTS], urinary tract infection [UTI]) in Groups I and II were comparable (LUTS, I vs II, 9.3% vs 12.7%, *P* = .366; UTI, 2.7% vs 2.0%, *P* = .561) (Table 2). UTI requiring hospitalization with antibiotics was reported in 1 patient in Group II and 2 patients in Group I. All 3 patients reported a full recovery. Severe rectal bleeding after prostate biopsy was recorded in 1 patient in Group II and 2 patients in Group I. All patients were hospitalized and managed with TRUS-guided compression using an ultrasound probe.

**Table 2:**
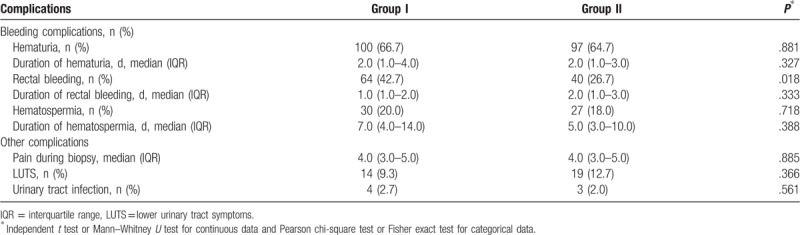
Complications after transrectal ultrasound-guided prostate biopsy.

Univariate analysis revealed a strong correlation between rectal bleeding rate after prostate biopsy and ORCP insertion (*P* = .018). Multivariate logistic regression analysis showed that ORCP insertion (*P* = .038, odds ratio OR 0.52) was significant protective factor against rectal bleeding after prostate biopsy (Table 3).

**Table 3:**
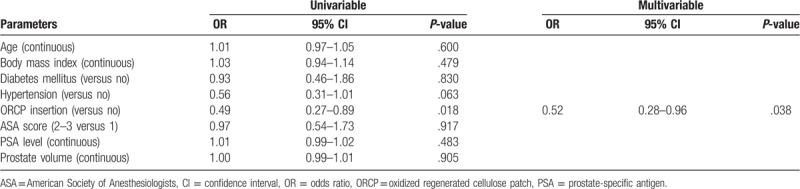
Logistic regression analysis for predictors of rectal bleeding after transrectal ultrasound-guided prostate biopsy.

**Figure 2:**
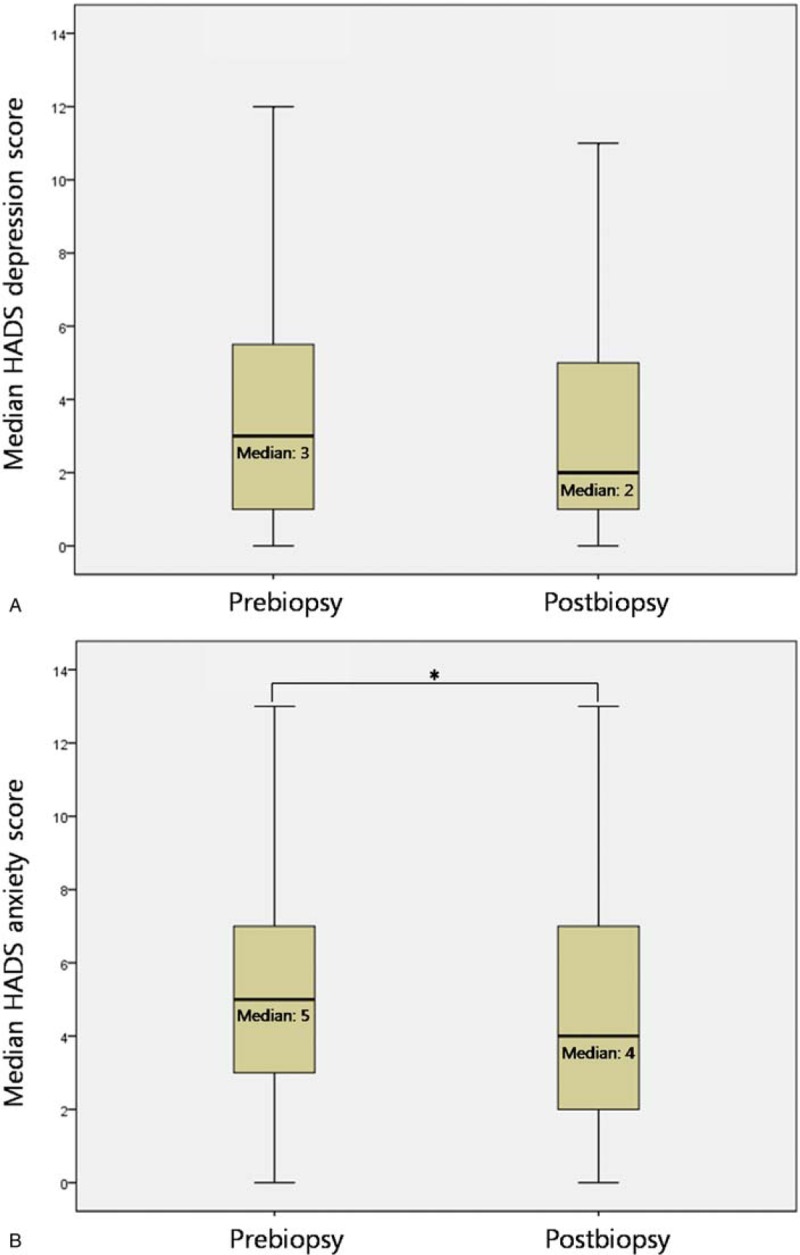
Box plot showing the HADS scores before transrectal ultrasound-guided prostate biopsy and after transrectal ultrasound-guided prostate biopsy and immediate insertion of oxidized regenerated cellulose patch into the rectum: A. Depression, B. Anxiety. Asterisk indicates *P* < .05. HADS = hospital anxiety and depression scale.

There was no statistically significant difference in median HADS depression scores before versus after prostate biopsy (3 vs 2, *P* = .648) (Fig. 2A). However, the difference between median HADS anxiety scores before and after prostate biopsy was statistically significant (5 vs 4, *P* = .011) (Fig. 2B).

## 4 Discussion

In this study of patients who underwent TRUS-guided biopsy of the prostate for histological diagnosis of PCa, we found that immediate insertion of ORCP presented significant hemostasis for rectal bleeding after prostate biopsy.

Gross hematuria following prostate biopsy is common, ranging from 2% to 84%.^[13,14]^ The frequency of hematuria in the present study was 66.7% in Group I and 64.7% in Group II. In our study, the median duration for hematuria was 2.0 days, which is compatible with results of 2.0 to 5.1 days in other published studies.^[5,15]^ The incidence rate of hematospermia reported in the literature varies widely (1.1%–92.6%).^[13,14]^ The frequency of hematospermia in the current study was 20% in Group I and 18% in Group II. In our study, the median duration for hematospermia was 5.0 to 7.0 days, which is similar to other study data of 2.0 to 20.0 days.^[16,17]^

Though clinically serious postbiopsy bleeding is rare, it can surprise unprepared patients. Moreover, life-threatening or massive rectal bleeding requires hospitalization or intervention. The incidence of rectal bleeding after prostate biopsy varies from 1.3% to 58.6%.^[14,18]^ The rectal bleeding frequency in the present study was 42.7% in Group I and 26.7% in Group II. The relatively high rates of rectal bleeding in the current study could be attributed to different methods of assessing rectal bleeding rate, differences in the definition of rectal bleeding, selection bias during patient recruitment, methods of data collection, and patient concern about rectal bleeding due to informed consent before the procedure.^[5,19]^ In the present study, the median duration for rectal bleeding was 1.0 to 2.0 days, which agrees with the 1.0 to 2.7 days reported in the literature.^[5,15]^ Regardless of the precise source of rectal bleeding, after prostate biopsy, the anterior rectal wall is the classical site of bleeding.^[18]^ ORCP is a natural, plant-derived topical absorbable hemostatic product that provides a surface for adhesion and aggregation of platelets. When placed on the anterior rectal wall following prostate biopsy, ORCP reacts with blood to form an artificial coagulant that acts as a substrate for further clotting. Moreover, its acidic property induces localized vasoconstriction, allowing ORCP to serve as a hemostatic adjunct.^[20,21]^ In addition, ORCP swells after application and could produce pressure on the site of rectal bleeding.^[22]^ Therefore, rectal bleeding frequency after prostate biopsy decreased in Group II.

The median VAS score during prostate biopsy in the current study was 4.0, which is comparable with that in published literature.^[14,23]^ Our rates of LUTS and UTI were 9.3% to 12.7% and 2% to 2.7%, respectively, similar to the incidence in published reports.^[14,24]^

We showed that ORCP insertion (*P* = .038, OR 0.52) was a significant, independent predictor of postbiopsy rectal bleeding. Our current findings show the significance of ORCP application as an important preventive factor for rectal bleeding after prostate biopsy. Kobatake et al^[4]^ found that insertion of a gelatin sponge into the rectum after prostate biopsy increased the hemostasis of rectal bleeding without increasing patient symptoms. However, insertion of a gelatin sponge in that study was performed without any guidance (eg, finger, TRUS) into the needle puncture site. Park et al^[5]^ compared ultrasound-guided compression on bleeding biopsy tracts immediately after TRUS-guided prostate biopsy versus a noncompression group and noted that the incidence of rectal bleeding was significantly lower in the ultrasound-guided compression group. However, there were patient symptoms, such as pain or discomfort for 5 to 10 minutes of compression via ultrasound transducer. To our knowledge, this is the first study to report that immediate ORCP insertion after prostate biopsy provides significant hemostasis for postbiopsy rectal bleeding.

Prostate biopsy can be a stressful procedure for patients. Previous studies regarding the psychological impact of prostate biopsy have reported variable results ranging from no apparent impact to most patients experiencing anxiety.^[25,26]^ Various strategies have been suggested to reduce the psychological distress of the procedure. Wade et al^[26]^ noted that accurate prebiopsy counseling and reassurance of the normality of some side effects (eg, hematuria, hematospermia) after prostate biopsy reduced the psychological distress (anxiety) caused by side effects. Chiu et al^[27]^ found that the combination of one-by-one simulation education and music therapy reduced anxiety for patients undergoing prostate biopsy. In this study, there was no significant difference between prebiopsy and postbiopsy HADS depression scores. However, the decrease in HADS anxiety score after immediate ORCP insertion following prostate biopsy in Group II was statistically significant. The median HADS anxiety score significantly decreased to 4 when immediate ORCP insertion occurred after prostate biopsy, suggesting that this strategy was effective. Our study is the first to reveal that ORCP insertion performed for decreasing postbiopsy bleeding complications reduces the anxiety level of patient undergoing TRUS-guided prostate biopsy.

Our study had some limitations. First, it was a comparison study of 2 groups in different time periods and had a nonrandomized nature. Second, this study used a nonvalidated questionnaire to assess postbiopsy complications.

## 5 Conclusions

This study demonstrated that immediate ORCP insertion after prostate biopsy significantly decreased the frequency of rectal bleeding but did not affect other postbiopsy bleeding complications. Therefore, ORCP insertion is an easy-to-use, useful, and practical method for decreasing postbiopsy rectal bleeding. Also, we found that ORCP insertion could reduce the anxiety level of patient undergoing prostate biopsy. Additional large, prospective randomized, controlled studies with adequately validated questionnaire are necessary to confirm our findings and provide solid data for evidence-based recommendations.

## Author contributions

Study concepts and design was done by BH Park, JW Park.

Acquisition of data and analysis of data was done by SR Bae, YS Lee, and SH Kang.

Analysis and interpretation of data was done by BH Park, JI Kim, and JW Park.

Manuscript preparation was done by BH Park and JW Park.

Manuscript editing was done by JI Kim and BH Park.

**Conceptualization:** Chang Hee Han, Bong Hee Park.

**Data curation:** Ji Woon Park, Bong Hee Park.

**Formal analysis:** Ji Woon Park, Jung Im Kim, Yong Seok Lee.

**Investigation:** Sang Rak Bae, Bong Hee Park.

**Methodology:** Jung Im Kim, Sang Rak Bae, Chang Hee Han, Sung Hak Kang, Bong Hee Park.

**Project administration:** Bong Hee Park.

**Supervision:** Chang Hee Han.

**Validation:** Yong Seok Lee, Sung Hak Kang.

**Writing – original draft:** Ji Woon Park.

**Writing – review and editing:** Jung Im Kim, Bong Hee Park.
